# Tris[*N*,*N*-bis­(3,5-di-*tert*-butyl­benz­yl)di­thio­carbamato-κ^2^ *S*,*S*′]-μ_3_-sulfido-tris-μ_2_-disulfido-*triangulo*-trimolybdenum(IV) iodide

**DOI:** 10.1107/S2414314620009396

**Published:** 2020-07-17

**Authors:** Yueli Chen, Bo Wang, Patricia Fontenot, James P. Donahue

**Affiliations:** a Department of Chemistry, University of Pennsylvania, 231 S. 34 Street, Philadelphia, PA 19104-6323, USA; b Department of Chemistry, Tulane University, 6400 Freret Street, New Orleans, Louisiana 70118-5698, USA; University of Kentucky, USA

**Keywords:** crystal structure, molybdenum-sulfide cluster, bulky di­thio­carbamate anion, dispersion forces

## Abstract

The title compound, [Mo_3_(C_31_H_46_NS_2_)_3_S_7_]I, crystallizes on a threefold rotational axis in *P*31*c* (space group No. 159). The [Mo_3_S_7_(S_2_CN(CH_2_C_6_H_3_-3,5-^*t*^Bu_2_)_2_)_3_]^+^ cations are arrayed in sheets in the *ab* plane with inter­ligand hydro­phobic inter­actions between *tert*-butyl groups guiding the packing arrangement. These cations form stacks parallel to the *c* axis with a separating distance of 10.9815 (6) Å (the *c* axis length) between the Mo_3_ centroids. On the underside of the cluster, opposite the μ_3_-S^2−^ ligand, the iodide counteranion forms close contacts of 3.166 (2) Å with the sulfur atoms of the μ_2_-S_2_^2−^ ligands. These contacts are less than the sum of the van der Waals radii of the atoms (1.8 and 2.1 Å for S and I, respectively), thus indicating an appreciable degree of covalency to the [Mo_3_S_7_(S_2_CN(CH_2_C_6_H_3_-3,5-^*t*^Bu_2_)_2_)_3_]^+^⋯I^−^ inter­actions.

## Abstract 2

[Mo_3_S_7_(S_2_CN(CH_2_C_6_H_3_-3,5-^*t*^Bu_2_)_2_)_3_]^+^⋯I^−^ crystallizes upon a threefold symmetry axis in *P*31*c* with the packing arrangement enforced by dispersion forces between the hydro­carbon-rich peripheral CH_2_C_6_H_3_-3,5-^*t*^Bu_2_ groups.

## Graphical abstract

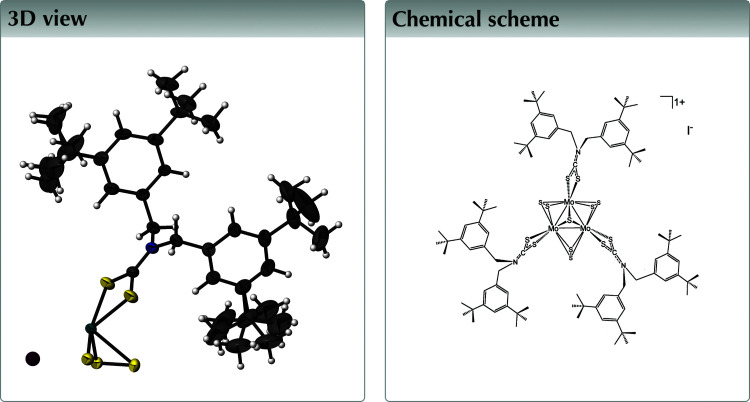

## Structure description

In a recent report (Fontenot *et al.*, 2019 ▸), we described photocatalytic H_2_ evolution by [Mo_3_S_7_(S_2_CN^*i*^Bu_2_)_3_]I in a MeCN/H_2_O mixture with [Ru(bipy)_3_]^2+^ as photosensitizer and Et_3_N as sacrificial electron donor. After a brief incubation period under photoylsis, the [Mo_3_S_7_(S_2_CN^*i*^Bu_2_)_3_]I cluster gives way to a charge-neutral asymmetric hexa­molybdenum species, comprised of [Mo_3_S_7_]^4+^ and [Mo_3_S_4_]^4+^ fragments, that appears to be the operative H_2_-evolution catalyst. As part of our efforts to understand solution speciation of [Mo_3_S_7_(S_2_CN*R*_2_)_3_]I (*R* = alk­yl) under photolysis, we endeavored to prepare and structurally characterize [Mo_3_S_4_(S_2_CN*R*_2_)_3_]^+^ clusters. Although stable to air and moisture and protracted handling, [Mo_3_S_4_(S_2_CN*R*_2_)_3_]^+^ clusters have proven to be surprisingly intractable to crystallization by typical methods. On the supposition that di­thio­carbamate supporting ligands with sufficiently large substituents would decisively dictate the solubility and crystallinity of a cluster to which they coordin­ate, we have prepared [Mo_3_S_7_(S_2_CN(CH_2_C_6_H_3_-3,5-^*t*^Bu_2_)_2_)_3_]I as a precursor to [Mo_3_S_4_(S_2_CN(CH_2_C_6_H_3_-3,5-^*t*^Bu_2_)_2_)_3_]^+^. In this communication, we briefly describe the crystal structure of [Mo_3_S_7_(S_2_CN(CH_2_C_6_H_3_-3,5-^*t*^Bu_2_)_2_)_3_]I.

The procedure implemented for the synthesis of [Mo_3_S_7_(S_2_CN(CH_2_C_6_H_3_-3,5-^*t*^Bu_2_)_2_)_3_]I is similar to that reported for [Mo_3_S_7_(S_2_CN^*i*^Bu_2_)_3_]I (Fontenot *et al.*, 2019 ▸). Although nominally an ion pair, [Mo_3_S_7_(S_2_CN(CH_2_C_6_H_3_-3,5-^*t*^Bu_2_)_2_)_3_]I is so dominated by the hydro­phobicity of the CH_2_C_6_H_3_-3,5-^*t*^Bu_2_ di­thio­carbamate substituents that it is readily taken up into C_6_H_6_. Addition of MeOH to a C_6_H_6_ solution to the point of incipient precipitation, followed by slow cooling, produces well-formed orange column-shaped crystals of [Mo_3_S_7_(S_2_CN(CH_2_C_6_H_3_-3,5-^*t*^Bu_2_)_2_)_3_]I with no inter­stitial solvent.

**Figure 1:**
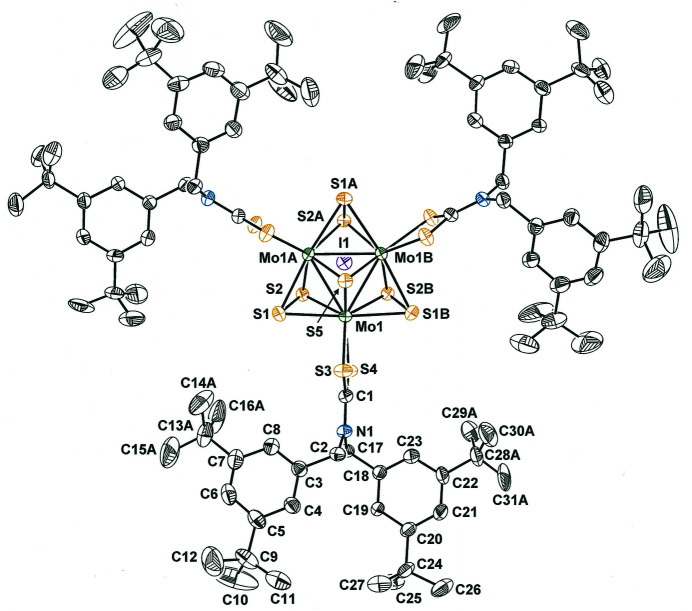
Displacement ellipsoid plot of [Mo_3_S_7_(S_2_CN(CH_2_C_6_H_3_-3,5-^*t*^Bu_2_)_2_)_3_]I at the 50% probability level with view along the threefold symmetry axis. All hydrogen atoms are omitted for clarity. *tert*-Butyl groups C13–C16 and C28–C31 are edited to show one of two positional variants in a split-atom model for the disorder. Symmetry codes: A = 1 − *y*, 1 + *x* − *y*, *z*; B = −*x* + *y*, 1 − *x*, *z*.

The triangular [Mo_3_S_7_(S_2_CN(CH_2_C_6_H_3_-3,5-^*t*^Bu_2_)_2_)_3_]^+^ cation coincides with a crystallographic threefold rotational axis, defined by S5 and the center of the Mo_3_ equilateral triangle (Fig. 1 ▸), in the non-centrosymmetric trigonal space group *P*31*c* (space group No. 159). Among the fair number of structural studies of [Mo_3_S_7_(S_2_CN*R*_2_)_3_]^+^·*X* ^−^ salts (*X* ^−^ = halide anion) that have been described (Zimmermann *et al.*, 1991 ▸; Fedin *et al.*, 1992 ▸, 1993 ▸; Lu *et al.*, 1993 ▸; Wang *et al.*, 1994 ▸; Mayor-López *et al.*, 1998 ▸; Il’inchik *et al.*, 2002 ▸, 2007 ▸; Fontenot *et al.*, 2019 ▸), in only one other instance (Fedin *et al.*, 1993 ▸) has the cluster cation been found on a threefold special position: [Mo_3_S_7_(S_2_CNEt_2_)_3_]I·1.5C_6_H_6_ in *R* 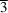 *c* (space group No. 167). The 3,5-^*t*^Bu_2_C_6_H_3_CH_2_ groups from each di­thio­carbamate ligand that are *syn* to the μ_3_-S^2−^ ligand (carbon atoms C1–C16) define a right-handed propeller when the cation is viewed from its ‘top’, or from the direction of the μ_3_-S^2−^ ligand toward the Mo_3_ centroid (Fig. 1 ▸). The remaining three 3,5-^*t*^Bu_2_C_6_H_3_CH_2_ groups (C17–C31), one from each ligand, are situated just below the Mo_3_ plane and define a left-handed propeller.

**Figure 2:**
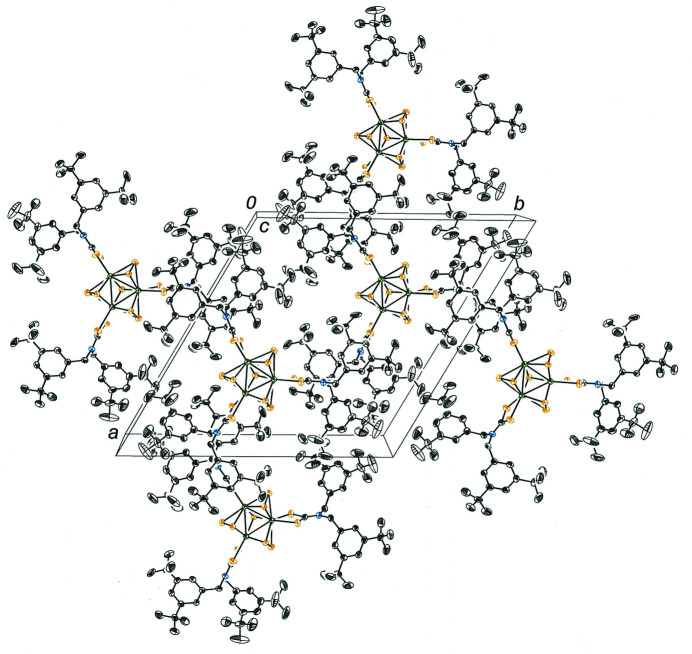
Cell packing diagram for the [Mo_3_S_7_(S_2_CN(CH_2_C_6_H_3_-3,5-^*t*^Bu_2_)_2_)_3_]^+^ cations showing the numerous inter­ligand hydro­phobic inter­actions. The view is down the *c* axis. All hydrogen atoms are omitted for clarity.

The occurrence of the trigonal space group for [Mo_3_S_7_(S_2_CN(CH_2_C_6_H_3_-3,5-^*t*^Bu_2_)_2_)_3_]I is guided by hydro­phobic inter­actions among the ^*t*^Bu groups of adjoining (3,5-^*t*^Bu_2_C_6_H_3_CH_2_)_2_NCS_2_^1–^ ligands from neighboring [Mo_3_S_7_(S_2_CN(CH_2_C_6_H_3_-3,5-^*t*^Bu_2_)_2_)_3_]^+^ cations (Fig. 2 ▸). The dispersion forces between these numerous hydro­carbon groups enforce an arrangement of [Mo_3_S_7_(S_2_CN(CH_2_C_6_H_3_-3,5-^*t*^Bu_2_)_2_)_3_]^+^ cations into sheets in the *ab* plane and stacking of these cations, one upon another, along the *c* axis with a separation equal to the *c-*axis length of 10.9815 (6) Å between adjacent Mo_3_ triangles. Inter­ligand π-stacking inter­actions with the benzyl groups are not effectively made due to the encumbering steric profile of the ^*t*^Bu groups.

**Figure 3:**
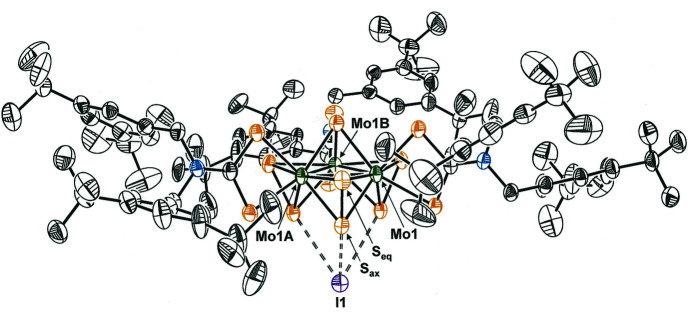
Side view of [Mo_3_S_7_(S_2_CN(CH_2_C_6_H_3_-3,5-^*t*^Bu_2_)_2_)_3_]I (50% probability for the displacement ellipsoids) showing the proximity of the I^−^ anion to the S_ax_ atoms of the μ_2_-S_2_^2−^ ligands.

The I^−^ counteranion is positioned on the underside of the [Mo_3_S_7_(S_2_CN(CH_2_C_6_H_3_-3,5-^*t*^Bu_2_)_2_)_3_]^+^ cation opposite the μ_3_-S^2−^ ligand (S5) (Fig. 3 ▸). A pronounced soft, electrophilic character to the S_axial_ atoms (Fig. 3 ▸) provides for close S_ax_⋯I^−^ contacts of 3.166 (2) Å, which are considerably below the sum of the van der Waals radii of the two atoms, 1.8 and 2.1 Å, respectively (Batsanov, 2001 ▸). Other distinctive structural features of [Mo_3_S_7_(S_2_CN(CH_2_C_6_H_3_-3,5-^*t*^Bu_2_)_2_)_3_]I are Mo—S_eq_ bond lengths [2.4847 (16) Å] that are appreciably longer by ∼0.080 Å than the Mo—S_ax_ [2.4056 (16) Å] bond lengths (Fig. 3 ▸), a longer Mo—S_di­thio­carbamate_ bond length for the sulfur atom that is *anti* to the μ_3_-S^2−^ ligand [2.5123 (16) Å] compared to the one that is *syn* [2.4816 (17) Å], and near orthogonality between the Mo_3_ plane and the S_2_CN chelate of the di­thio­carbamate ligand. These parameters are quite similar to those observed in related compounds (Fontenot *et al.*, 2019 ▸).

In continuing work, we target the deliberate synthesis of hexa­molybdenum sulfide clusters by fusion of separate [Mo_3_S_7_]^4+^ and [Mo_3_S_4_]^4+^ fragments.

## Synthesis and crystallization

[NH_4_]_2_[Mo_3_S_13_] and (3,5-^*t*^Bu_2_–C_6_H_3_CH_2_)_2_NC(S)S–SC(S)N(CH_2_C_6_H_3_-3,5-^*t*^Bu_2_)_2_ were reacted in a 1:3 ratio following a procedure detailed earlier (Fontenot *et al.*, 2019 ▸). Crystallization was accomplished by layering MeOH onto a solution of the title compound in C_6_H_6_ and cooling the set-up to −20°C for 48 h.

## Refinement

Crystal data, data collection and structure refinement details are summarized in Table 1 ▸. The *tert*-butyl groups defined by C13–C16 and C28–C31 are disordered and were treated with independent, floating site occupancy variables that identified 0.687 (13):0.313 (13) and 0.623 (11):0.377 (11) optimal partitioning, respectively, for the two groups.

## Supplementary Material

Crystal structure: contains datablock(s) I, global. DOI: 10.1107/S2414314620009396/pk4027sup1.cif

Structure factors: contains datablock(s) I. DOI: 10.1107/S2414314620009396/pk4027Isup3.hkl

CCDC reference: 2015330

Additional supporting information:  crystallographic information; 3D view; checkCIF report

## Figures and Tables

**Table 1:** Experimental details

Crystal data	
Chemical formula	[Mo_3_(C_31_H_46_NS_2_)_3_S_7_]I
*M*_r_	2129.56
Crystal system, space group	Trigonal, *P*31*c*
Temperature (K)	150
*a*, *c* (Å)	23.6627 (12), 10.9815 (6)
*V* (Å^3^)	5325.0 (6)
*Z*	2
Radiation type	Mo *K*α
μ (mm^−1^)	0.93
Crystal size (mm)	0.38 × 0.16 × 0.13

Data collection	
Diffractometer	Bruker SMART APEX CCD
Absorption correction	Multi-scan (*SADABS*; Krause *et al*., 2015 ▸)
*T*_min_, *T*_max_	0.799, 0.890
No. of measured, independent and observed [*I* > 2σ(*I*)] reflections	89878, 7810, 7053
*R*_int_	0.043
(sin θ/λ)_max_ (Å^−1^)	0.641

Refinement	
*R*[*F* ^2^ > 2σ(*F* ^2^)], *wR*(*F* ^2^), *S*	0.037, 0.104, 1.06
No. of reflections	7810
No. of parameters	369
No. of restraints	35
H-atom treatment	H-atom parameters constrained
Δρ_max_, Δρ_min_ (e Å^−3^)	1.08, −0.54
Absolute structure	Flack *x* determined using 3209 quotients [(*I* ^+^)−(*I* ^−^)]/[(*I* ^+^)+(*I* ^−^)] (Parsons *et al*., 2013 ▸)
Absolute structure parameter	0.014 (8)

Computer programs: *APEX3* and *SAINT* (Bruker, 2016 ▸), *SHELXT/5* (Sheldrick, 2015*a* ▸), *SHELXL2018/3* (Sheldrick, 2015*b* ▸) and *SHELXTL* (Sheldrick, 2008 ▸).

## full crystallographic data

### Crystal data

**Table:**

[Mo_3_(C_31_H_46_NS_2_)_3_S_7_]I	*D*_x_ = 1.328 Mg m^−^^3^
*M**_r_* = 2129.56	Mo *K*α radiation, λ = 0.71073 Å
Trigonal, *P*31*c*	Cell parameters from 9927 reflections
*a* = 23.6627 (12) Å	θ = 2.8–27.0°
*c* = 10.9815 (6) Å	µ = 0.93 mm^−^^1^
*V* = 5325.0 (6) Å^3^	*T* = 150 K
*Z* = 2	Column, yellow-orange
*F*(000) = 2208	0.38 × 0.16 × 0.13 mm

### Data collection

**Table:**

Bruker SMART APEX CCD diffractometer	7810 independent reflections
Radiation source: fine-focus sealed tube	7053 reflections with *I* > 2σ(*I*)
Graphite monochromator	*R*_int_ = 0.043
Detector resolution: 8.3333 pixels mm^-1^	θ_max_ = 27.1°, θ_min_ = 1.7°
φ and ω scans	*h* = −30→30
Absorption correction: multi-scan (*SADABS*; Krause et al., 2015)	*k* = −30→30
*T*_min_ = 0.799, *T*_max_ = 0.890	*l* = −14→14
89878 measured reflections	

### Refinement

**Table:**

Refinement on *F*^2^	Secondary atom site location: difference Fourier map
Least-squares matrix: full	Hydrogen site location: inferred from neighbouring sites
*R*[*F*^2^ > 2σ(*F*^2^)] = 0.037	H-atom parameters constrained
*wR*(*F*^2^) = 0.104	*w* = 1/[σ^2^(*F*_o_^2^) + (0.0543*P*)^2^ + 6.2404*P*] where *P* = (*F*_o_^2^ + 2*F*_c_^2^)/3
*S* = 1.06	(Δ/σ)_max_ < 0.001
7810 reflections	Δρ_max_ = 1.08 e Å^−^^3^
369 parameters	Δρ_min_ = −0.54 e Å^−^^3^
35 restraints	Absolute structure: Flack *x* determined using 3209 quotients [(*I*^+^)-(*I*^-^)]/[(*I*^+^)+(*I*^-^)] (Parsons et al., 2013)
Primary atom site location: structure-invariant direct methods	Absolute structure parameter: 0.014 (8)

### Special details

**Table:**

Experimental. The diffraction data were obtained from 3 sets of 400 frames, each of width 0.5° in ω, collected at φ = 0.00, 90.00 and 180.00° and 2 sets of 800 frames, each of width 0.45° in φ, collected at ω = –30.00 and 210.00°. The scan time was 60 sec/frame.
Geometry. All esds (except the esd in the dihedral angle between two l.s. planes) are estimated using the full covariance matrix. The cell esds are taken into account individually in the estimation of esds in distances, angles and torsion angles; correlations between esds in cell parameters are only used when they are defined by crystal symmetry. An approximate (isotropic) treatment of cell esds is used for estimating esds involving l.s. planes.
Refinement. Refinement of F^2^ against ALL reflections. The weighted R-factor wR and goodness of fit S are based on F^2^, conventional R-factors R are based on F, with F set to zero for negative F^2^. The threshold expression of F^2^ > 2sigma(F^2^) is used only for calculating R-factors(gt) etc. and is not relevant to the choice of reflections for refinement. R-factors based on F^2^ are statistically about twice as large as those based on F, and R- factors based on ALL data will be even larger. H-atoms attached to carbon were placed in calculated positions (C—H = 0.95 - 0.99 Å). All were included as riding contributions with isotropic displacement parameters 1.2 - 1.5 times those of the attached atoms.

### Fractional atomic coordinates and isotropic or equivalent isotropic displacement parameters (Å^2^)

**Table:**

	*x*	*y*	*z*	*U*_iso_*/*U*_eq_	Occ. (<1)
I1	0.333333	0.666667	0.90377 (7)	0.04082 (19)	
Mo1	0.26950 (2)	0.59847 (2)	0.52052 (5)	0.02818 (12)	
S1	0.34151 (8)	0.55022 (7)	0.50720 (16)	0.0357 (3)	
S2	0.33840 (7)	0.58992 (7)	0.67175 (15)	0.0319 (3)	
S3	0.20057 (8)	0.51946 (8)	0.36144 (15)	0.0376 (3)	
S4	0.17377 (8)	0.50221 (8)	0.61825 (15)	0.0367 (3)	
S5	0.333333	0.666667	0.3562 (3)	0.0324 (6)	
N1	0.0983 (2)	0.4120 (2)	0.4549 (5)	0.0313 (10)	
C1	0.1493 (3)	0.4695 (3)	0.4762 (6)	0.0329 (13)	
C2	0.0818 (3)	0.3809 (3)	0.3340 (6)	0.0349 (13)	
H2A	0.106606	0.413943	0.270705	0.042*	
H2B	0.034714	0.362867	0.318052	0.042*	
C3	0.0980 (3)	0.3270 (3)	0.3274 (6)	0.0359 (13)	
C4	0.0558 (3)	0.2684 (3)	0.2686 (6)	0.0387 (14)	
H4	0.015801	0.262103	0.236242	0.046*	
C5	0.0710 (4)	0.2194 (3)	0.2564 (7)	0.0448 (16)	
C6	0.1312 (4)	0.2316 (4)	0.3071 (7)	0.0500 (18)	
H6	0.143434	0.199243	0.297989	0.060*	
C7	0.1722 (3)	0.2870 (4)	0.3679 (7)	0.0490 (17)	
C8	0.1555 (3)	0.3358 (3)	0.3757 (6)	0.0409 (15)	
H8	0.184360	0.375826	0.415063	0.049*	
C9	0.0279 (4)	0.1560 (4)	0.1908 (7)	0.059 (2)	
C10	−0.0007 (7)	0.1002 (5)	0.2846 (11)	0.158 (9)	
H10A	−0.028735	0.058766	0.242989	0.237*	
H10B	0.034978	0.098041	0.325727	0.237*	
H10C	−0.026364	0.108271	0.344856	0.237*	
C11	−0.0264 (5)	0.1557 (5)	0.1227 (11)	0.096 (4)	
H11A	−0.052421	0.113449	0.082352	0.144*	
H11B	−0.054124	0.163012	0.179441	0.144*	
H11C	−0.008584	0.190495	0.061491	0.144*	
C12	0.0675 (6)	0.1397 (6)	0.1009 (11)	0.108 (5)	
H12A	0.038426	0.098479	0.059666	0.161*	
H12B	0.088215	0.174704	0.040398	0.161*	
H12C	0.101238	0.135673	0.145355	0.161*	
C13A	0.2382 (5)	0.3013 (7)	0.4211 (12)	0.063 (3)	0.687 (13)
C14A	0.2939 (7)	0.3563 (8)	0.3491 (15)	0.089 (5)	0.687 (13)
H14A	0.291089	0.343326	0.263623	0.133*	0.687 (13)
H14B	0.290458	0.395848	0.354580	0.133*	0.687 (13)
H14C	0.335819	0.365089	0.383072	0.133*	0.687 (13)
C15A	0.2454 (9)	0.2409 (8)	0.4133 (18)	0.114 (7)	0.687 (13)
H15A	0.242288	0.227403	0.328066	0.171*	0.687 (13)
H15B	0.287876	0.250929	0.446377	0.171*	0.687 (13)
H15C	0.210561	0.205388	0.460566	0.171*	0.687 (13)
C16A	0.2430 (10)	0.3218 (10)	0.5527 (12)	0.109 (9)	0.687 (13)
H16A	0.207406	0.286758	0.599318	0.163*	0.687 (13)
H16B	0.284964	0.330617	0.586254	0.163*	0.687 (13)
H16C	0.239602	0.361376	0.557762	0.163*	0.687 (13)
C13B	0.2345 (10)	0.2935 (16)	0.426 (2)	0.063 (3)	0.313 (13)
C14B	0.2834 (15)	0.298 (2)	0.330 (3)	0.089 (5)	0.313 (13)
H14D	0.263069	0.258144	0.279677	0.133*	0.313 (13)
H14E	0.296254	0.335955	0.278445	0.133*	0.313 (13)
H14F	0.322046	0.301406	0.370474	0.133*	0.313 (13)
C15B	0.2197 (17)	0.2363 (18)	0.510 (3)	0.114 (7)	0.313 (13)
H15D	0.197541	0.195506	0.463729	0.171*	0.313 (13)
H15E	0.260638	0.241905	0.543413	0.171*	0.313 (13)
H15F	0.191562	0.234837	0.576852	0.171*	0.313 (13)
C16B	0.269 (2)	0.3560 (18)	0.501 (4)	0.109 (9)	0.313 (13)
H16D	0.239241	0.355362	0.564161	0.163*	0.313 (13)
H16E	0.307768	0.359213	0.539869	0.163*	0.313 (13)
H16F	0.281976	0.393762	0.447841	0.163*	0.313 (13)
C17	0.0557 (3)	0.3703 (3)	0.5544 (6)	0.0347 (13)	
H17A	0.074882	0.390668	0.633795	0.042*	
H17B	0.052960	0.327200	0.551264	0.042*	
C18	−0.0116 (3)	0.3613 (3)	0.5439 (6)	0.0342 (13)	
C19	−0.0634 (3)	0.3013 (3)	0.5099 (6)	0.0332 (12)	
H19	−0.056448	0.265524	0.496737	0.040*	
C20	−0.1259 (3)	0.2928 (3)	0.4947 (7)	0.0412 (15)	
C21	−0.1349 (3)	0.3460 (4)	0.5215 (9)	0.055 (2)	
H21	−0.177392	0.340490	0.515471	0.066*	
C22	−0.0829 (4)	0.4066 (3)	0.5568 (11)	0.066 (3)	
C23	−0.0215 (3)	0.4128 (3)	0.5669 (8)	0.0485 (18)	
H23	0.014534	0.453648	0.590186	0.058*	
C24	−0.1834 (3)	0.2258 (4)	0.4600 (7)	0.0456 (17)	
C25	−0.2046 (4)	0.1810 (4)	0.5663 (8)	0.057 (2)	
H25A	−0.220352	0.198209	0.630869	0.085*	
H25B	−0.167545	0.177373	0.596736	0.085*	
H25C	−0.239714	0.137779	0.541574	0.085*	
C26	−0.2412 (4)	0.2316 (5)	0.4121 (9)	0.065 (2)	
H26A	−0.272334	0.190832	0.371604	0.098*	
H26B	−0.225367	0.267717	0.353849	0.098*	
H26C	−0.262758	0.239912	0.480275	0.098*	
C27	−0.1633 (5)	0.1952 (5)	0.3569 (9)	0.065 (2)	
H27A	−0.129055	0.186834	0.386317	0.097*	
H27B	−0.146708	0.225365	0.287630	0.097*	
H27C	−0.201220	0.154079	0.331081	0.097*	
C28A	−0.0935 (6)	0.4631 (6)	0.6020 (13)	0.067 (5)	0.623 (11)
C29A	−0.0620 (8)	0.4899 (8)	0.7275 (13)	0.093 (6)	0.623 (11)
H29A	−0.015827	0.502399	0.724929	0.139*	0.623 (11)
H29B	−0.065853	0.528240	0.747614	0.139*	0.623 (11)
H29C	−0.084441	0.456222	0.789649	0.139*	0.623 (11)
C30A	−0.0590 (8)	0.5175 (8)	0.5091 (15)	0.089 (6)	0.623 (11)
H30A	−0.013143	0.528904	0.502718	0.133*	0.623 (11)
H30B	−0.080166	0.502716	0.429608	0.133*	0.623 (11)
H30C	−0.061350	0.555839	0.535113	0.133*	0.623 (11)
C31A	−0.1645 (6)	0.4449 (10)	0.6127 (17)	0.075 (5)	0.623 (11)
H31A	−0.186393	0.409848	0.672707	0.113*	0.623 (11)
H31B	−0.167145	0.483117	0.638956	0.113*	0.623 (11)
H31C	−0.185962	0.429994	0.533451	0.113*	0.623 (11)
C28B	−0.0926 (11)	0.4657 (8)	0.5325 (19)	0.067 (5)	0.377 (11)
C29B	−0.1503 (13)	0.4527 (18)	0.613 (2)	0.093 (6)	0.377 (11)
H29D	−0.141602	0.444973	0.697197	0.139*	0.377 (11)
H29E	−0.156563	0.490635	0.611492	0.139*	0.377 (11)
H29F	−0.189823	0.414213	0.583465	0.139*	0.377 (11)
C30B	−0.0323 (12)	0.5275 (12)	0.577 (3)	0.089 (6)	0.377 (11)
H30D	0.005206	0.536402	0.526147	0.133*	0.377 (11)
H30E	−0.040157	0.564348	0.572735	0.133*	0.377 (11)
H30F	−0.023174	0.521408	0.661902	0.133*	0.377 (11)
C31B	−0.1068 (12)	0.4775 (12)	0.4032 (18)	0.075 (5)	0.377 (11)
H31D	−0.069929	0.485906	0.350528	0.113*	0.377 (11)
H31E	−0.146372	0.438987	0.373704	0.113*	0.377 (11)
H31F	−0.113112	0.515410	0.401730	0.113*	0.377 (11)

### Atomic displacement parameters (Å^2^)

**Table:**

	*U*^11^	*U*^22^	*U*^33^	*U*^12^	*U*^13^	*U*^23^
I1	0.0389 (3)	0.0389 (3)	0.0447 (4)	0.01945 (13)	0.000	0.000
Mo1	0.0219 (2)	0.0211 (2)	0.0392 (3)	0.00897 (19)	−0.0007 (2)	−0.0010 (2)
S1	0.0356 (8)	0.0283 (7)	0.0477 (9)	0.0192 (7)	0.0003 (6)	−0.0037 (6)
S2	0.0279 (7)	0.0260 (7)	0.0432 (8)	0.0145 (6)	0.0011 (6)	0.0024 (6)
S3	0.0318 (8)	0.0290 (7)	0.0398 (8)	0.0060 (6)	0.0003 (6)	−0.0005 (6)
S4	0.0302 (7)	0.0291 (7)	0.0381 (8)	0.0054 (6)	−0.0006 (6)	0.0001 (6)
S5	0.0296 (8)	0.0296 (8)	0.0380 (13)	0.0148 (4)	0.000	0.000
N1	0.025 (2)	0.027 (2)	0.037 (3)	0.009 (2)	0.002 (2)	0.002 (2)
C1	0.025 (3)	0.028 (3)	0.043 (3)	0.012 (2)	−0.001 (2)	0.000 (2)
C2	0.032 (3)	0.028 (3)	0.041 (3)	0.012 (3)	−0.004 (2)	−0.002 (2)
C3	0.039 (3)	0.035 (3)	0.035 (3)	0.019 (3)	−0.002 (3)	0.000 (2)
C4	0.042 (4)	0.034 (3)	0.042 (4)	0.020 (3)	−0.002 (3)	0.000 (3)
C5	0.060 (4)	0.033 (3)	0.045 (4)	0.026 (3)	0.005 (3)	0.005 (3)
C6	0.060 (5)	0.048 (4)	0.058 (4)	0.039 (4)	0.009 (4)	0.008 (3)
C7	0.047 (4)	0.056 (5)	0.055 (4)	0.033 (4)	0.005 (3)	0.007 (3)
C8	0.038 (4)	0.043 (4)	0.045 (4)	0.022 (3)	−0.001 (3)	−0.002 (3)
C9	0.086 (6)	0.034 (4)	0.053 (4)	0.027 (4)	0.003 (4)	0.000 (3)
C10	0.22 (2)	0.050 (7)	0.097 (10)	−0.007 (9)	−0.010 (11)	0.009 (7)
C11	0.090 (8)	0.057 (6)	0.135 (10)	0.032 (6)	−0.033 (8)	−0.046 (7)
C12	0.130 (11)	0.124 (11)	0.109 (9)	0.095 (10)	−0.030 (8)	−0.056 (8)
C13A	0.062 (6)	0.092 (8)	0.061 (5)	0.058 (6)	−0.005 (4)	0.003 (5)
C14A	0.044 (7)	0.111 (12)	0.104 (11)	0.033 (9)	0.007 (7)	0.025 (11)
C15A	0.101 (14)	0.144 (16)	0.145 (19)	0.097 (14)	−0.031 (13)	0.001 (16)
C16A	0.115 (18)	0.20 (3)	0.081 (14)	0.13 (2)	−0.037 (12)	−0.038 (13)
C13B	0.062 (6)	0.092 (8)	0.061 (5)	0.058 (6)	−0.005 (4)	0.003 (5)
C14B	0.044 (7)	0.111 (12)	0.104 (11)	0.033 (9)	0.007 (7)	0.025 (11)
C15B	0.101 (14)	0.144 (16)	0.145 (19)	0.097 (14)	−0.031 (13)	0.001 (16)
C16B	0.115 (18)	0.20 (3)	0.081 (14)	0.13 (2)	−0.037 (12)	−0.038 (13)
C17	0.030 (3)	0.025 (3)	0.044 (3)	0.010 (2)	−0.001 (2)	0.004 (2)
C18	0.028 (3)	0.030 (3)	0.044 (3)	0.014 (3)	0.002 (2)	0.007 (3)
C19	0.026 (3)	0.030 (3)	0.044 (3)	0.015 (3)	−0.002 (2)	0.001 (2)
C20	0.028 (3)	0.035 (3)	0.056 (4)	0.012 (3)	−0.007 (3)	0.009 (3)
C21	0.025 (3)	0.038 (4)	0.100 (7)	0.015 (3)	0.001 (4)	0.010 (4)
C22	0.040 (4)	0.031 (4)	0.134 (8)	0.024 (3)	0.004 (5)	0.008 (4)
C23	0.029 (3)	0.032 (3)	0.079 (5)	0.011 (3)	0.001 (3)	0.004 (3)
C24	0.023 (3)	0.044 (4)	0.062 (4)	0.011 (3)	−0.006 (3)	0.012 (3)
C25	0.049 (4)	0.043 (4)	0.057 (5)	0.007 (4)	−0.005 (3)	0.009 (3)
C26	0.037 (4)	0.064 (5)	0.077 (6)	0.012 (4)	−0.016 (4)	0.019 (4)
C27	0.053 (5)	0.054 (5)	0.067 (6)	0.011 (4)	−0.008 (4)	−0.009 (4)
C28A	0.052 (6)	0.039 (5)	0.123 (17)	0.034 (5)	0.009 (10)	0.011 (9)
C29A	0.084 (11)	0.069 (9)	0.144 (17)	0.051 (9)	−0.014 (10)	−0.041 (10)
C30A	0.096 (14)	0.055 (8)	0.131 (19)	0.049 (10)	0.012 (11)	0.028 (11)
C31A	0.091 (11)	0.077 (10)	0.095 (11)	0.070 (10)	0.027 (9)	0.044 (8)
C28B	0.052 (6)	0.039 (5)	0.123 (17)	0.034 (5)	0.009 (10)	0.011 (9)
C29B	0.084 (11)	0.069 (9)	0.144 (17)	0.051 (9)	−0.014 (10)	−0.041 (10)
C30B	0.096 (14)	0.055 (8)	0.131 (19)	0.049 (10)	0.012 (11)	0.028 (11)
C31B	0.091 (11)	0.077 (10)	0.095 (11)	0.070 (10)	0.027 (9)	0.044 (8)

### Geometric parameters (Å, º)

**Table:**

I1—S2	3.1657 (17)	C14B—H14E	0.9800
Mo1—S5	2.389 (2)	C14B—H14F	0.9800
Mo1—S2	2.4056 (16)	C15B—H15D	0.9800
Mo1—S2^i^	2.4096 (16)	C15B—H15E	0.9800
Mo1—S1^i^	2.4801 (16)	C15B—H15F	0.9800
Mo1—S3	2.4816 (17)	C16B—H16D	0.9800
Mo1—S1	2.4847 (16)	C16B—H16E	0.9800
Mo1—S4	2.5123 (16)	C16B—H16F	0.9800
Mo1—Mo1^ii^	2.7100 (8)	C17—C18	1.501 (9)
Mo1—Mo1^i^	2.7100 (8)	C17—H17A	0.9900
S1—S2	2.055 (2)	C17—H17B	0.9900
S3—C1	1.738 (7)	C18—C23	1.375 (10)
S4—C1	1.708 (7)	C18—C19	1.385 (9)
N1—C1	1.313 (8)	C19—C20	1.399 (9)
N1—C2	1.472 (8)	C19—H19	0.9500
N1—C17	1.479 (8)	C20—C21	1.407 (11)
C2—C3	1.505 (9)	C20—C24	1.535 (10)
C2—H2A	0.9900	C21—C22	1.399 (11)
C2—H2B	0.9900	C21—H21	0.9500
C3—C8	1.376 (10)	C22—C23	1.391 (10)
C3—C4	1.397 (9)	C22—C28A	1.558 (11)
C4—C5	1.381 (9)	C22—C28B	1.548 (12)
C4—H4	0.9500	C23—H23	0.9500
C5—C6	1.419 (11)	C24—C25	1.486 (10)
C5—C9	1.510 (10)	C24—C26	1.533 (10)
C6—C7	1.354 (11)	C24—C27	1.542 (12)
C6—H6	0.9500	C25—H25A	0.9800
C7—C8	1.397 (10)	C25—H25B	0.9800
C7—C13A	1.539 (11)	C25—H25C	0.9800
C7—C13B	1.545 (13)	C26—H26A	0.9800
C8—H8	0.9500	C26—H26B	0.9800
C9—C11	1.483 (10)	C26—H26C	0.9800
C9—C10	1.540 (9)	C27—H27A	0.9800
C9—C12	1.538 (10)	C27—H27B	0.9800
C10—H10A	0.9800	C27—H27C	0.9800
C10—H10B	0.9800	C28A—C31A	1.518 (11)
C10—H10C	0.9800	C28A—C30A	1.520 (11)
C11—H11A	0.9800	C28A—C29A	1.544 (12)
C11—H11B	0.9800	C29A—H29A	0.9800
C11—H11C	0.9800	C29A—H29B	0.9800
C12—H12A	0.9800	C29A—H29C	0.9800
C12—H12B	0.9800	C30A—H30A	0.9800
C12—H12C	0.9800	C30A—H30B	0.9800
C13A—C16A	1.510 (12)	C30A—H30C	0.9800
C13A—C14A	1.531 (12)	C31A—H31A	0.9800
C13A—C15A	1.524 (12)	C31A—H31B	0.9800
C14A—H14A	0.9800	C31A—H31C	0.9800
C14A—H14B	0.9800	C28B—C29B	1.526 (13)
C14A—H14C	0.9800	C28B—C31B	1.518 (13)
C15A—H15A	0.9800	C28B—C30B	1.527 (13)
C15A—H15B	0.9800	C29B—H29D	0.9800
C15A—H15C	0.9800	C29B—H29E	0.9800
C16A—H16A	0.9800	C29B—H29F	0.9800
C16A—H16B	0.9800	C30B—H30D	0.9800
C16A—H16C	0.9800	C30B—H30E	0.9800
C13B—C16B	1.525 (14)	C30B—H30F	0.9800
C13B—C14B	1.534 (14)	C31B—H31D	0.9800
C13B—C15B	1.524 (14)	C31B—H31E	0.9800
C14B—H14D	0.9800	C31B—H31F	0.9800
			
S5—Mo1—S2	110.63 (5)	H16A—C16A—H16B	109.5
S5—Mo1—S2^i^	110.49 (5)	C13A—C16A—H16C	109.5
S2—Mo1—S2^i^	85.04 (8)	H16A—C16A—H16C	109.5
S5—Mo1—S1^i^	85.44 (4)	H16B—C16A—H16C	109.5
S2—Mo1—S1^i^	134.46 (6)	C16B—C13B—C14B	106.8 (12)
S2^i^—Mo1—S1^i^	49.68 (6)	C16B—C13B—C15B	108.1 (12)
S5—Mo1—S3	86.13 (6)	C14B—C13B—C15B	107.7 (12)
S2—Mo1—S3	129.83 (6)	C16B—C13B—C7	110 (2)
S2^i^—Mo1—S3	134.34 (6)	C14B—C13B—C7	111.9 (19)
S1^i^—Mo1—S3	92.23 (6)	C15B—C13B—C7	112 (2)
S5—Mo1—S1	85.33 (4)	C13B—C14B—H14D	109.5
S2—Mo1—S1	49.66 (6)	C13B—C14B—H14E	109.5
S2^i^—Mo1—S1	134.40 (6)	H14D—C14B—H14E	109.5
S1^i^—Mo1—S1	170.77 (7)	C13B—C14B—H14F	109.5
S3—Mo1—S1	87.47 (6)	H14D—C14B—H14F	109.5
S5—Mo1—S4	156.09 (6)	H14E—C14B—H14F	109.5
S2—Mo1—S4	88.32 (5)	C13B—C15B—H15D	109.5
S2^i^—Mo1—S4	84.78 (5)	C13B—C15B—H15E	109.5
S1^i^—Mo1—S4	91.49 (6)	H15D—C15B—H15E	109.5
S3—Mo1—S4	70.28 (5)	C13B—C15B—H15F	109.5
S1—Mo1—S4	97.08 (5)	H15D—C15B—H15F	109.5
S5—Mo1—Mo1^ii^	55.44 (4)	H15E—C15B—H15F	109.5
S2—Mo1—Mo1^ii^	55.82 (4)	C13B—C16B—H16D	109.5
S2^i^—Mo1—Mo1^ii^	96.44 (4)	C13B—C16B—H16E	109.5
S1^i^—Mo1—Mo1^ii^	116.88 (4)	H16D—C16B—H16E	109.5
S3—Mo1—Mo1^ii^	126.34 (4)	C13B—C16B—H16F	109.5
S1—Mo1—Mo1^ii^	56.84 (4)	H16D—C16B—H16F	109.5
S4—Mo1—Mo1^ii^	143.68 (4)	H16E—C16B—H16F	109.5
S5—Mo1—Mo1^i^	55.44 (4)	N1—C17—C18	111.0 (5)
S2—Mo1—Mo1^i^	96.54 (4)	N1—C17—H17A	109.4
S2^i^—Mo1—Mo1^i^	55.68 (4)	C18—C17—H17A	109.4
S1^i^—Mo1—Mo1^i^	57.00 (4)	N1—C17—H17B	109.4
S3—Mo1—Mo1^i^	129.44 (4)	C18—C17—H17B	109.4
S1—Mo1—Mo1^i^	116.72 (4)	H17A—C17—H17B	108.0
S4—Mo1—Mo1^i^	139.31 (4)	C23—C18—C19	120.2 (6)
Mo1^ii^—Mo1—Mo1^i^	60.0	C23—C18—C17	119.9 (6)
S2—S1—Mo1^ii^	63.38 (6)	C19—C18—C17	119.9 (6)
S2—S1—Mo1	63.17 (6)	C18—C19—C20	120.6 (6)
Mo1^ii^—S1—Mo1	66.16 (4)	C18—C19—H19	119.7
S1—S2—Mo1	67.17 (6)	C20—C19—H19	119.7
S1—S2—Mo1^ii^	66.95 (6)	C19—C20—C21	117.9 (6)
Mo1—S2—Mo1^ii^	68.50 (5)	C19—C20—C24	120.4 (6)
S1—S2—I1	172.02 (8)	C21—C20—C24	121.5 (6)
Mo1—S2—I1	106.60 (5)	C20—C21—C22	121.8 (7)
Mo1^ii^—S2—I1	106.50 (5)	C20—C21—H21	119.1
C1—S3—Mo1	88.5 (2)	C22—C21—H21	119.1
C1—S4—Mo1	88.2 (2)	C23—C22—C21	117.8 (6)
Mo1^i^—S5—Mo1	69.12 (7)	C23—C22—C28A	119.4 (8)
Mo1^i^—S5—Mo1^ii^	69.12 (7)	C21—C22—C28A	122.2 (8)
Mo1—S5—Mo1^ii^	69.12 (7)	C23—C22—C28B	122.5 (10)
C1—N1—C2	123.7 (5)	C21—C22—C28B	115.3 (11)
C1—N1—C17	121.8 (5)	C18—C23—C22	121.6 (7)
C2—N1—C17	114.3 (5)	C18—C23—H23	119.2
N1—C1—S4	124.1 (5)	C22—C23—H23	119.2
N1—C1—S3	122.9 (5)	C25—C24—C20	110.5 (6)
S4—C1—S3	113.0 (3)	C25—C24—C26	109.7 (6)
N1—C2—C3	110.6 (5)	C20—C24—C26	111.3 (7)
N1—C2—H2A	109.5	C25—C24—C27	108.4 (7)
C3—C2—H2A	109.5	C20—C24—C27	110.1 (6)
N1—C2—H2B	109.5	C26—C24—C27	106.8 (7)
C3—C2—H2B	109.5	C24—C25—H25A	109.5
H2A—C2—H2B	108.1	C24—C25—H25B	109.5
C8—C3—C4	119.4 (6)	H25A—C25—H25B	109.5
C8—C3—C2	120.2 (6)	C24—C25—H25C	109.5
C4—C3—C2	120.4 (6)	H25A—C25—H25C	109.5
C5—C4—C3	121.4 (7)	H25B—C25—H25C	109.5
C5—C4—H4	119.3	C24—C26—H26A	109.5
C3—C4—H4	119.3	C24—C26—H26B	109.5
C4—C5—C6	116.6 (7)	H26A—C26—H26B	109.5
C4—C5—C9	123.4 (7)	C24—C26—H26C	109.5
C6—C5—C9	120.0 (7)	H26A—C26—H26C	109.5
C7—C6—C5	123.4 (7)	H26B—C26—H26C	109.5
C7—C6—H6	118.3	C24—C27—H27A	109.5
C5—C6—H6	118.3	C24—C27—H27B	109.5
C6—C7—C8	118.0 (7)	H27A—C27—H27B	109.5
C6—C7—C13A	123.8 (8)	C24—C27—H27C	109.5
C8—C7—C13A	118.1 (8)	H27A—C27—H27C	109.5
C6—C7—C13B	119.1 (14)	H27B—C27—H27C	109.5
C8—C7—C13B	122.9 (14)	C31A—C28A—C30A	110.0 (9)
C3—C8—C7	121.1 (6)	C31A—C28A—C29A	106.5 (10)
C3—C8—H8	119.4	C30A—C28A—C29A	107.8 (10)
C7—C8—H8	119.4	C31A—C28A—C22	114.3 (12)
C5—C9—C11	113.5 (7)	C30A—C28A—C22	105.3 (10)
C5—C9—C10	108.7 (7)	C29A—C28A—C22	112.7 (10)
C11—C9—C10	109.0 (8)	C28A—C29A—H29A	109.5
C5—C9—C12	111.2 (8)	C28A—C29A—H29B	109.5
C11—C9—C12	108.1 (7)	H29A—C29A—H29B	109.5
C10—C9—C12	106.0 (8)	C28A—C29A—H29C	109.5
C9—C10—H10A	109.5	H29A—C29A—H29C	109.5
C9—C10—H10B	109.5	H29B—C29A—H29C	109.5
H10A—C10—H10B	109.5	C28A—C30A—H30A	109.5
C9—C10—H10C	109.5	C28A—C30A—H30B	109.5
H10A—C10—H10C	109.5	H30A—C30A—H30B	109.5
H10B—C10—H10C	109.5	C28A—C30A—H30C	109.5
C9—C11—H11A	109.5	H30A—C30A—H30C	109.5
C9—C11—H11B	109.5	H30B—C30A—H30C	109.5
H11A—C11—H11B	109.5	C28A—C31A—H31A	109.5
C9—C11—H11C	109.5	C28A—C31A—H31B	109.5
H11A—C11—H11C	109.5	H31A—C31A—H31B	109.5
H11B—C11—H11C	109.5	C28A—C31A—H31C	109.5
C9—C12—H12A	109.5	H31A—C31A—H31C	109.5
C9—C12—H12B	109.5	H31B—C31A—H31C	109.5
H12A—C12—H12B	109.5	C29B—C28B—C31B	108.9 (12)
C9—C12—H12C	109.5	C29B—C28B—C30B	108.6 (12)
H12A—C12—H12C	109.5	C31B—C28B—C30B	108.1 (11)
H12B—C12—H12C	109.5	C29B—C28B—C22	104 (2)
C16A—C13A—C14A	109.0 (10)	C31B—C28B—C22	118.3 (15)
C16A—C13A—C15A	109.3 (10)	C30B—C28B—C22	108.7 (16)
C14A—C13A—C15A	108.5 (10)	C28B—C29B—H29D	109.5
C16A—C13A—C7	109.8 (11)	C28B—C29B—H29E	109.5
C14A—C13A—C7	109.7 (10)	H29D—C29B—H29E	109.5
C15A—C13A—C7	110.5 (11)	C28B—C29B—H29F	109.5
C13A—C14A—H14A	109.5	H29D—C29B—H29F	109.5
C13A—C14A—H14B	109.5	H29E—C29B—H29F	109.5
H14A—C14A—H14B	109.5	C28B—C30B—H30D	109.5
C13A—C14A—H14C	109.5	C28B—C30B—H30E	109.5
H14A—C14A—H14C	109.5	H30D—C30B—H30E	109.5
H14B—C14A—H14C	109.5	C28B—C30B—H30F	109.5
C13A—C15A—H15A	109.5	H30D—C30B—H30F	109.5
C13A—C15A—H15B	109.5	H30E—C30B—H30F	109.5
H15A—C15A—H15B	109.5	C28B—C31B—H31D	109.5
C13A—C15A—H15C	109.5	C28B—C31B—H31E	109.5
H15A—C15A—H15C	109.5	H31D—C31B—H31E	109.5
H15B—C15A—H15C	109.5	C28B—C31B—H31F	109.5
C13A—C16A—H16A	109.5	H31D—C31B—H31F	109.5
C13A—C16A—H16B	109.5	H31E—C31B—H31F	109.5

Symmetry codes: (i) −*x*+*y*, −*x*+1, *z*; (ii) −*y*+1, *x*−*y*+1, *z*.
